# Comparison of the Anticancer Effects of a Complementary Peptide for Dickkopf WNT Signaling Pathway Inhibitor 3 (DKK3) With Conventional Anticancer Drugs in the Treatment of Oral Squamous Cell Carcinoma: A Pilot Study

**DOI:** 10.7759/cureus.91625

**Published:** 2025-09-04

**Authors:** Naoki Katase, Shin-ichiro Nishimatsu, Akira Yamauchi, Shuichi Fujita

**Affiliations:** 1 Department of Oral Pathology, Graduate School of Biomedical Sciences, Nagasaki University, Nagasaki, JPN; 2 Department of Natural Sciences, Kawasaki Medical School, Kurashiki, JPN; 3 Department of Biochemistry, Kawasaki Medical School, Kurashiki, JPN

**Keywords:** anticancer agent, complementary peptide, dickkopf wnt signaling pathway inhibitor 3 (dkk3), oral cancers, oral squamous cell carcinoma, squamous cell carcinoma (scc)

## Abstract

Introduction

Oral squamous cell carcinoma (OSCC), which is the most common cancer type in head and neck cancers, remains a serious health problem because of its high mortality. Treatment of OSCC is mainly performed with a combination of surgery and anticancer agents. However, despite the recent development of anticancer agents, the clinical outcome of OSCC has yet to be improved. Therefore, it is required to develop a new anticancer agent that targets a specific molecular target in OSCC. In this context, we identified DKK3 (Dickkopf WNT signaling pathway inhibitor 3) as a candidate for such targets. Our previous studies have demonstrated that DKK3 expression is observed in certain cancers, such as OSCC and esophageal squamous cell carcinoma, and DKK3 increases tumor malignancy through Akt activation in such cancers, while its expression is lost in many kinds of malignancies. Recently, we developed a complementary peptide targeting DKK3 (DKK3-CP) as a new anticancer substance and reported that DKK3-CP could significantly suppress cancer cell growth, migration, and invasion in OSCC-derived cells. In the present research, we compared the anticancer effects of the peptide with conventional anticancer agents as a pilot study.

Methods

OSCC-derived cell lines (HSC-3 and SAS) were treated with cisplatin (CDDP), cetuximab (Cmab), or DKK3-CP at various concentrations. As a control, saline was added to the cells, reproducing the no-treatment condition. The effects on cellular viability, cellular migration, and invasion were investigated. The differences within the groups were compared, and the differences between the groups at certain concentrations were compared by one-way ANOVA with Tukey's multiple comparisons. An orthotopic xenograft model was used for the evaluation of systemic administration of the agents. HSC-3 cells were orthotopically transplanted into the tongue of the mice, and after confirming tumor formation, anticancer agents were administered intraperitoneally. Saline was used for control to reproduce the no-treatment condition. Then, the animals were sacrificed and evaluated histologically. The Ki-67 index was also calculated.

Results

DKK3-CP showed significant suppressive effects for cell viability at 10 µM in both cell lines. In HSC-3 cells, there was no significant difference in the suppressive effect on cell viability among the anticancer agents, while the suppressive effects of DKK3-CP were significantly higher than that of CDDP in SAS cells. Migration and invasion assays revealed that DKK3-CP showed significant suppression of migration and invasion at 500 nM in both cell lines, and the effect was significantly higher than that of CDDP and Cmab. Systemic administration of DKK3-CP did not show weight loss in the mice. Histological evaluation revealed that all anticancer agents significantly decreased the Ki-67 index, although no significant difference was observed between those groups.

Conclusion

DKK3-CP showed tumor suppression effects comparable or superior to those of CDDP and Cmab. Although this is a pilot study and ideal concentration and administration methods of the agents, combination use of DKK3-CP with conventional agents, appropriate evaluation of side effects, and assessment of the long-term effects of administration should be resolved by further investigations, we showed the possibility of clinical use of DKK3-CP for the first time.

## Introduction

Oral squamous cell carcinoma (OSCC) is the most common form of head and neck squamous cell carcinoma (HNSCC). A recent report revealed that more than 377,000 new cases of OSCC are diagnosed annually, and its incidence is higher in countries with low and medium human development index [1]. OSCC may arise from any part of the squamous epithelia in the oral cavity and includes quite heterogeneous lesions with different oncogenic initiating events and distinct genetic profiles. OSCC shows significant chromosomal alterations and high somatic mutation burden [2], but the key genetic and/or epigenetic alterations are not well explained. The survival rate of OSCC is not favorable; the five-year survival rate in the early stages is up to 72%, but it becomes significantly worse at less than 50% in advanced stages [3-5].

The main treatment strategy for OSCC is surgery, supported by anticancer agents and radiation therapy [6,7]. Surgical excision of OSCC in the tongue, gingiva with jaw bones, and teeth may cause difficulty in speech and food intake, and consequent decline in quality of life. Anticancer agents used in OSCC treatment are sometimes cytotoxic, and their outcome is still not improved enough. For example, cisplatin (cis-diamminedichloroplatinum: CDDP), a well-known chemotherapeutic reagent, interferes with the DNA repair mechanism and causes DNA damage and consequent cancer cell apoptosis. It is reported that postoperative radiotherapy combined with CDDP therapy significantly reduces local and regional recurrences and prolongs disease-free survival (DFS) in patients with advanced OSCC, but CDDP has problems such as drug resistance and side effects, including kidney damage and gastrointestinal disorder [8,9]. Cetuximab (Cmab), a monoclonal antibody that inhibits epidermal growth factor receptor (EGFR), appears to be associated with tumor growth inhibition and is one of the few targeted therapies currently used [10-12]. However, OSCC cases with the activation of the PI3K signaling pathway are reported to be associated with resistance to this treatment [10]; therefore, its individual administration may not be effective enough. Immune checkpoint inhibitors (ICIs) such as pembrolizumab or nivolumab give hope for unresectable OSCC. Some clinical trials revealed that nivolumab alone or in combination therapy showed favorable anti-tumor immune responses in OSCC, but at the moment, more studies are required for further confirmation of the efficacy and underlying mechanisms [13-15].

In this context, we tried to develop a new anticancer substance. We previously identified DKK3 (Dickkopf WNT signaling pathway inhibitor 3) as a candidate therapeutic target in HNSCC/OSCC [16]. The DKK3 gene encodes both secreted and non-secreted proteins that possess two distinct cysteine-rich domains (CRDs). DKK3 protein inhibits oncogenic WNT signaling and functions as a tumor suppressor by competing with Wnt ligands binding to its receptor, Frizzled and LRP5/6, or blocking nuclear translocation of beta-catenin [16,17]. Indeed, DKK3 protein expression is weakened or lost in many kinds of malignancies. However, some types of cancers, including HNSCC/OSCC and esophageal squamous cell carcinoma, specifically express DKK3, and in such cancers, DKK3 exerts oncogenic function via activation of Akt signaling [18]. Our series of research revealed oncogenic function of DKK3 in HNSCC/OSCC, and we developed complementary peptides that bind to the CRDs of DKK3 and inhibit the function of DKK3 [19-21]. The DKK3 complementary peptide (DKK3-CP) can inhibit cellular proliferation, migration, invasion, and *in vivo* tumor growth of the subcutaneously transplanted tumors with direct injection of DKK3-CP at low doses, and is thought to be a promising new therapeutic reagent. In this research, we compared the anticancer effects of the peptides with conventional anticancer reagents, including CDDP and Cmab, using OSCC-derived cell lines *in vitro *and *in vivo*. To apply DKK3-CP into clinical use, we needed to confirm whether it can also exert anticancer effects when it is administered systemically. Then, we chose the orthotopic tongue transplantation model as an *in vivo* model to reproduce the clinical condition of tongue cancer.

## Materials and methods

Cell lines and anticancer reagents

Human OSCC-derived cell lines (HSC-3 and SAS) were purchased from RIKEN BioResource Center (Tsukuba, Japan). Both HSC-3 and SAS originate from human tongue cancer, known to show high expression of EGFR [22]. HSC-3 cells were selected for the experiment due to their high metastatic potential and aggressive characteristics, which are also known as a key model for studying tongue cancer. Also, HSC-3 is commonly used in xenograft models in mice. HSC-3 cells secrete growth factors that promote angiogenesis and have a known mutation in the *TP53* gene. SAS cells were selected for the experiment due to their high growth rate and high metastatic potential. They are known to have mutations in the *TP53* gene, and are commonly used as a model for oral cancer research, including studies on chemoresistance.

HSC-3 cells were maintained in Dulbecco's modified Eagle's medium (DMEM; FUJIFILM Wako Pure Chemical Corporation, Osaka, Japan) and SAS cells were maintained in RPMI-1640 medium (FUJIFILM Wako Pure Chemical Corporation), supplemented with 10% fetal bovine serum (FBS; Cosmo Bio Co., Ltd., Tokyo, Japan) and Penicillin-Streptomycin-Amphotericin B Suspension (FUJIFILM Wako Pure Chemical Corporation), in an incubator with an atmosphere of 5% CO_2_ at 37°C.

CDDP (FUJIFILM Wako Pure Chemical Corporation) was suspended in PBS as a 200 mg/mL stock. Erbitux® (Merck KGaA, Darmstadt, Germany) was used as Cmab. DKK3-CP was synthesized as previously described (GenScript Japan Inc., Tokyo, Japan). DKK3-CP consists of two peptides targeting the two distinct functional domains (i.e., CRDs) of DKK3, namely, CRD1 and CRD2. The amino acid sequences of the peptides with oligoarginine (number of R = 12) for CRD1 and CRD2 are AVTVTWTLTATTTATTTLHTRRRRRRRRRRRR and APFQRYWQFTYWKLSLEWRRRRRRRRRRRR, respectively. The peptides were designed by an evolutionary software program, MIMETEC, which employs a genetic algorithm that generates a series of increasingly optimized peptides for a target by random alteration of amino acids for 5,000 generations. The docking of the peptides and CRDs of DKK3 was confirmed by docking simulation, and the effects of the peptides have been validated in our previous report [21]. DKK3-CP was diluted in PBS as a 4 mg/mL stock.

Since the design of complementary peptides is delicate and complex, it is impossible to predict how randomly designed peptide amino acid sequences will bind to other proteins and interact with them, making it extremely difficult to design peptides with arbitrary amino acid sequences as controls. Then, we set untreated cells (adding saline only) as the control in both *in vitro* and* in vivo *experiments, which was indicated as “no treatment” in the figures.

Cell viability

Cellular viability was assessed by CCK-8 assay (Dojindo, Kumamoto, Japan). Cells were seeded in 96-well plates in 1.0 × 10^3^ cells/100 µL/ well. The CCK-8 reagents were added and incubated with the cells for one hour. The absorbance at 450 nm was measured by a plate reader. Data were taken 24 hours after treatment with reagents. CDDP, Cmab, and DKK3-CP were added to the cells at final concentrations of 50 nM, 100 nM, 500 nM, 1 µM, and 10 µM. As a control, saline was added instead of the anticancer reagents. The experiment was performed in the same fashion in both HSC-3 cells and SAS cells.

The cell viability was calculated and compared in a relative value, setting "no treatment" as 1. The relative cell viability was calculated as follows. The actual measured values of the cell viability were recorded, and the actual average (A) was calculated. This value was then divided by the actual average of the control (no treatment) to calculate the relative average value (R). By multiplying each measured value by R and dividing it by A, the measured values were converted to relative values, and we confirmed that the average of these relative values matched R. The standard deviation was calculated from these relative values and plotted on a graph.

The effects on cellular viability were compared within the group in each concentration, and additionally, those within the group were compared at 10 µM.

Migration assay (wound healing assay)

The cellular migration was assessed as a wound healing assay, using an Ibidi Culture-Insert (Ibidi GmbH, Munich, Germany) according to the manufacturer's protocol. Cells were suspended in media supplemented with 10% FBS (1.0×10^6^ cells/ml), and 70 μL of cell suspension was transferred to each well of the Culture-Insert in a six-well plate. CDDP, Cmab, and DKK3-CP were added to the cells at final concentrations of 500 nM, 1 µM, and 10 µM. As a control, saline was added instead of the anticancer reagents. The experiment was performed in the same fashion in both HSC-3 cells and SAS cells.

After 24 hours of incubation at 37°C in an atmosphere containing 5% CO_2_, the Culture-Insert was removed using sterilized tweezers. Photos were taken using a microscope, soon after the removal of the Culture-Insert (at 0 hours) and six hours later. The area was measured using Image J software version 1.51 (http://rsb.info.nih.gov/ij/; National Institutes of Health, Bethesda, MD, USA).

Wound healing ratio was calculated by dividing the area at six hours by that at 0 hours. And then the wound healing ratio was compared in a relative value, setting the "no treatment" control group as 1. We calculated the wound healing ratio of each group, and the actual average (A) was calculated. This value was then divided by the actual average of the control (no treatment) to calculate the relative average value (R). By multiplying each measured value by R and dividing it by A, the measured values were converted to relative values, and we confirmed that the average of these relative values matched R. The standard deviation was calculated from these relative values and plotted on a graph.

The effects on cellular migration were compared within the group in each concentration, and additionally, those among the group were compared at 10 µM.

Matrigel invasion assay

BioCoat^TM^ Matrigel® Invasion Chambers (Corning Life Sciences, Bedford, MA, USA) were used to conduct an invasion assay. Cells were harvested and suspended in serum-free media at 2.5 × 10^5^ cells/ml, and 500 μL cell suspension was added to the upper chambers. CDDP, Cmab, and DKK3-CP were added to the cells at final concentrations of 500 nM, 1 µM, and 10 µM. As a control, saline was added instead of the anticancer reagents. The experiment was performed in the same fashion in both HSC-3 cells and SAS cells.

After 24 hours of incubation at 37°C in an atmosphere containing 5% CO_2_, the chambers were fixed and stained with Diff-Quik Stain^TM^ (Lab Aids Pty Ltd., North Narrabeen, Australia) and mounted on a glass slide. Cell number was counted under an optical microscope, and invasion index (% invasion) was calculated according to the manufacturer's protocol.

The effects on cellular invasion were compared within the group in each concentration, and additionally, those among the group were compared at 10 µM.

Orthotopic xenograft model and histological evaluation

This animal study was performed in accordance with the Guidelines for Animal Experiments at Nagasaki University, and the animal protocol for this study was approved by the Animal Care and Use Committee of Nagasaki University (No. 2304261857, 2023). Only HSC-3 cells were used in the xenograft model because of the known ability of tumor formation in nude mice of HSC-3.

HSC-3 cells were suspended in PBS at 1.0 × 10^5^ to 5.0 × 10^5 ^cells/25 µL DMEM and were injected into the right lateral portion of the tongue of five-week-old, male BALB/cAJcl-nu/nu nude mice (CLEA Japan, Inc., Tokyo, Japan). The animals had free access to food and water and were housed at 25°C (60-70 % humidity), under a 12-hour light/dark cycle. A total of 36 mice were used in this study, which were divided into four groups: (i) injected with HSC-3 cells and no treatment done (administration of saline only) (N = 11); (ii) HSC-3 + CDDP treatment (N = 7); (iii) HSC-3 + Cmab treatment (N = 7); and (iv) HSC-3 + DKK3-CP treatment (N = 11). On day seven after injection of tumor cells, we started treatment, and all the anticancer agents were intraperitoneally administered at 5 mg/kg/week.

For the assessment of side effects, the mice were weighed at days 0, 2, 5, 8, 11, 14, 17, 20, 23, and 26. The weight of the mice was compared in a relative value. Since there was variation in the weight of the mice and there was already a significant difference between groups at day 0, we calculated the change in weight as a relative, setting the average weight of each group at day 0 as 1. The relative weight was calculated as follows. The actual measured values of the body weights were recorded on each weighing day, and the actual average (A) was calculated. This value was then divided by the actual average of the weights on day 0 to calculate the relative average value (R) for each weighing day. By multiplying each measured value by R and dividing it by A, the measured values were converted to relative values, and we confirmed that the average of these relative values matched R. The standard deviation was calculated from these relative values and plotted on a graph.

On day 28, the mice were sacrificed, and tumors were collected for histological evaluation. Tissues were then fixed in 10% neutral buffered formalin for eight hours at room temperature, embedded in paraffin, sectioned at 4 μm, and stained with hematoxylin-eosin (HE). In addition, tissue sections underwent immunohistochemistry (IHC). IHC for Ki-67 was performed using Histofine® Simple Stain MAX PO (Multi) (Nichirei Biosciences Inc., Tokyo, Japan) and antibody against Ki-67 (27309-1-AP, Proteintech, Rosemont, IL, USA) according to the manufacturer’s instructions. Tissue sections were deparaffinized in xylene and then dehydrated in graded alcohol solutions. The endogenous peroxidase activity was blocked by immersing the sections in 0.3% H_2_O_2_ in methanol for 30 minutes. Antigen retrieval was done by heating sections in Tris-EDTA buffer (pH: 9.0) for 10 minutes. The sections were then treated with 10% normal serum for 30 minutes, followed by incubation with primary antibodies (1:15000) at 4°C overnight. Identification of an immunoreaction was achieved by subsequent incubation with a biotinylated secondary antibody for 30 minutes, followed by detection using the avidin-biotin complex. Sections were then reacted with TaKaRa DUB Substrate (Takara Bio Inc., Kusatsu, Japan). The sections were counterstained by Carrazzi's hematoxylin and mounted. We took photos of all five areas under a 20x lens and counted Ki-67 positive cells, and the Ki-67 labeling index was calculated.

Statistics

All values are presented as means ± standard deviation. Significant differences for cellular viability, migration, invasion, and Ki-67 index were determined using one-way ANOVA for multiple comparisons with Tukey's multiple comparisons. All analyses were conducted using R version 4.5.1 (R Foundation for Statistical Computing, Vienna, Austria; http://www.r-project.org/). The p-value <0.05 was considered to indicate a statistically significant difference.

## Results

Cell viability assay

**Figure 1:**
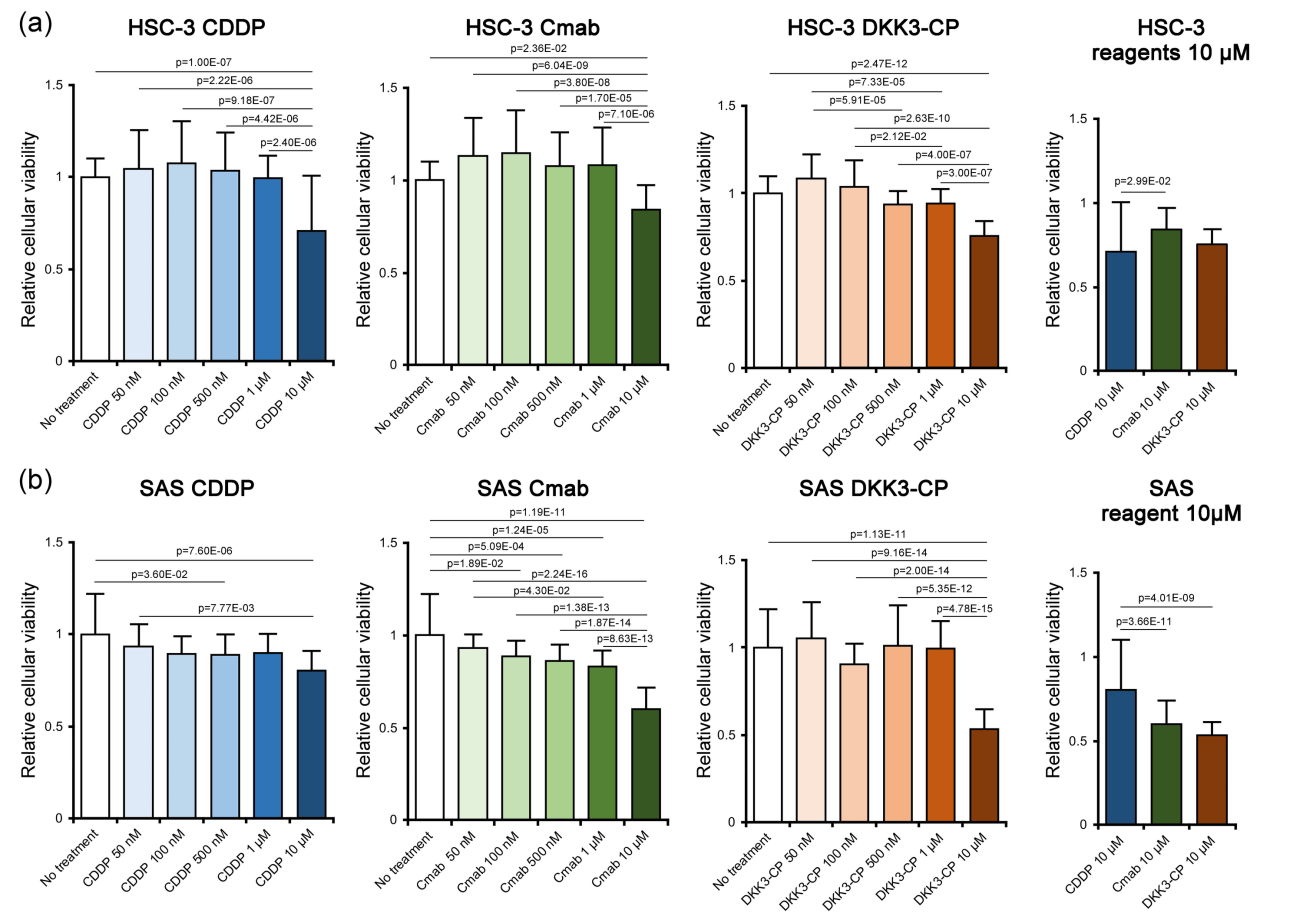
Cell viability assay. (a) In HSC-3 cells, all anticancer agents significantly suppressed cellular viability at 10 µM. Comparison among the groups at 10 µM revealed that CDDP showed significantly higher suppressive effects than Cmab, whereas there were no significant differences between CDDP and DKK3-CP or between Cmab and DKK3-CP. (b) In SAS cells, CDDP and Cmab showed significantly decreased cellular viability at 500 nM and 100 nM, respectively, while DKK3-CP showed suppressive effects for cellular viability at 10 µM. However, comparison among the groups at 10 µM revealed that DKK3-CP showed significantly higher effects than that of CDDP. CDDP: cisplatin; Cmab: cetuximab; DKK3-CP: DKK3 complementary peptide.

In HSC-3 cells, all the anticancer reagents significantly diminished cellular viability in a final concentration of 10 µM (CDDP: p = 1.00E-07; Cmab: p = 2.36E-02; and DKK3-CP: p = 2.47E-12). When the suppressive effects among the groups were compared at 10 µM, CDDP showed significantly higher suppressive effects than Cmab (p = 2.99E-02), whereas there were no significant differences between CDDP and DKK3-CP or between Cmab and DKK3-CP (Figure 1A).

In SAS cells, CDDP and Cmab showed significantly decreased cellular viability at 500 nM (p = 3.60E-02) and 100 nM (p = 1.89E-02), respectively, while DKK3-CP showed suppressive effects for cellular viability at 10 µM (p = 1.13E-11). However, comparison among the groups at 10 µM revealed that DKK3-CP showed significantly higher effects than that of CDDP (p = 4.01E-09) (Figure 1B).

Migration assay (wound healing assay)

**Figure 2:**
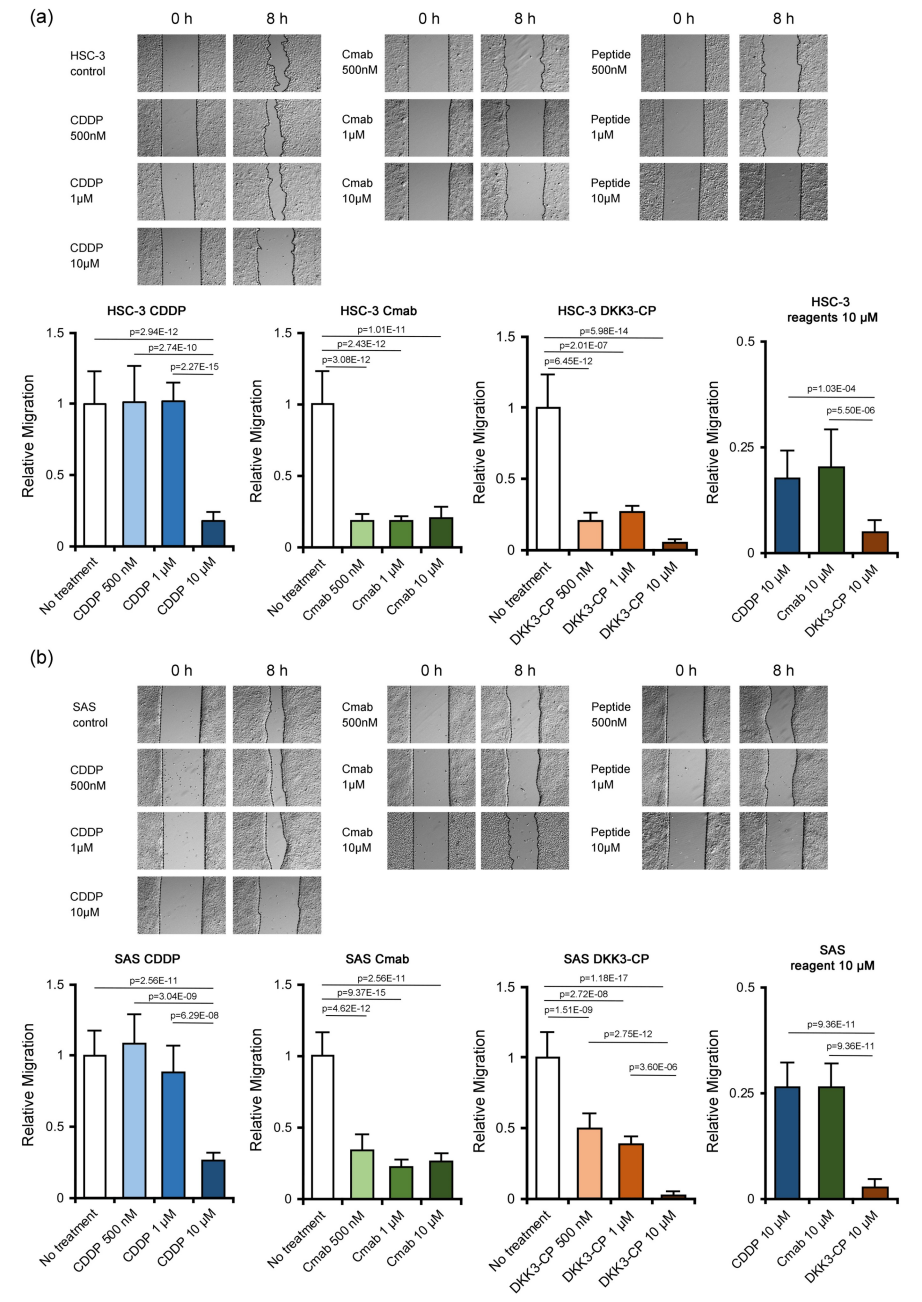
Migration assay. (a) In HSC-3 cells, CDDP did not show suppressive effects at 500 nM and 1 µM, while 10 µM showed a significant suppressive effect for migration. Cmab showed significant migration suppression at 500 nM, 1 µM, and 10 µM. Similar to Cmab, DKK3-CP showed significantly suppressed migration at 500 nM, 1 µM, and 10 µM. DKK3-CP showed more intense suppressive effects on cellular migration at 10 µM than CDDP or Cmab. (b) In SAS cells, CDDP could not suppress cellular migration at 500 nM and 1 µM, although it suppressed cellular migration at 10 µM. Cmab significantly suppressed cellular migration at 500 nM, 1 µM, and 10 µM. DKK3-CP also showed significant suppressive effects on migration at 500 nM, 1 µM, and 10 µM. The effect was significantly higher than that of CDDP and Cmab. CDDP: cisplatin; Cmab: cetuximab; DKK3-CP: DKK3 complementary peptide.

In HSC-3 cells, CDDP did not show suppressive effects at 500 nM and 1 µM, while 10 µM showed a significant suppressive effect for migration (p = 2.94E-12). Cmab showed significant migration suppression at 500 nM (p = 3.08E-12), 1 µM (p = 2.43E-12), and 10 µM (p = 1.01E-11). Similarly to Cmab, DKK3-CP showed significantly suppressed migration at 500 nM (p = 6.45E-12), 1 µM (p = 2.01E-7), and 10 µM (p = 5.98E-14). DKK3-CP showed more intense suppressive effects on cellular migration at 10 µM than CDDP (p = 1.03E-04) or Cmab (p = 5.50E-06) (Figure 2A).

In SAS cells, the results were similar to those of HSC-3 cells. CDDP could not suppress cellular migration at 500 nM and 1 µM, although it suppressed cellular migration at 10 µM (p = 2.56E-11). Cmab significantly suppressed cellular migration at 500 nM (p = 4.62E-12), 1 µM (p = 9.37E-15), and 10 µM (p = 2.56E-11), and DKK3-CP also showed significant suppressive effects on migration at 500 nM (p = 1.51E-09), 1 µM (p = 2.72E-08), and 10 µM (p = 1.18E-17). Notably, DKK3-CP could almost completely suppress cellular migration at 10 µM, and the effect was significantly higher than that of CDDP (p = 9.36E-11) and Cmab (p = 9.36E-11) (Figure 2B).

Matrigel invasion assay

**Figure 3:**
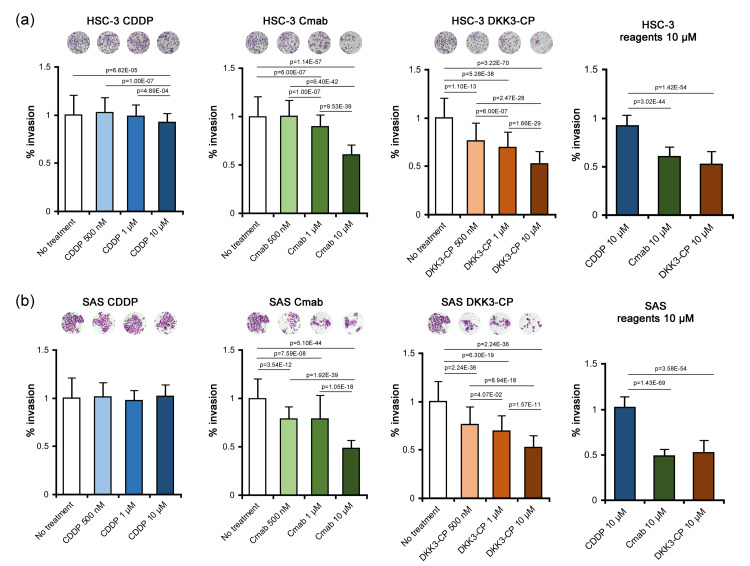
Invasion assay. (a) In HSC-3 cells, CDDP significantly suppressed invasion at 10 µM. Cmab significantly suppressed cellular invasion at 1 µM and 10 µM, while DKK3-CP showed suppressive effects on invasion at 500 nM, 1 µM, and 10 µM. Comparison among the groups at 10 µM showed that DKK3-CP showed significantly higher suppressive effect for invasion than CDDP. (b) In SAS cells, CDDP could not suppress cancer cell invasion at all. Cmab significantly suppressed invasion at 500 nM, 1 µM, and 10 µM. DKK3-CP suppressed invasion at 500 nM, 1 µM, and 10 µM. Similar to HSC-3, comparison among the groups at 10 µM showed that DKK3-CP showed significantly higher suppressive effect for invasion than CDDP. CDDP: cisplatin; Cmab: cetuximab; DKK3-CP: DKK3 complementary peptide.

In HSC-3 cells, the suppressive effects of CDDP on invasion were limited; significant suppression was observed only at 10 µM (p = 6.82E-05). Cmab significantly suppressed cellular invasion at 1 µM (p = 6.00E-07) and 10 µM (p = 1.14E-57). DKK3-CP showed suppressive effects on invasion at 500 nM (p = 1.10E-13), 1 µM (p = 5.28E-38), and 10 µM (p = 3.22E-70). Comparison among the groups at 10 µM showed that DKK3-CP showed significantly higher suppressive effect for invasion than CDDP (p = 1.42E-54) (Figure 3A).

In SAS cells, CDDP could not suppress cancer cell invasion at all. In Cmab treatment, the suppressive effect for invasion was observed at 500 nM (p = 3.54E-12), 1 µM (p = 7.59E-08), and 10 µM (p = 5.10E-44). DKK3-CP suppressed invasion at 500 nM (p = 2.24E-36), 1 µM (p = 6.30E-19), and 10 µM (p = 2.24E-36). Similarly to HSC-3, comparison among the group at 10 µM showed that DKK3-CP showed significantly higher suppressive effect for invasion than CDDP (p = 3.58E-54) (Figure 3B).

Orthotopic xenograft model

**Figure 4:**
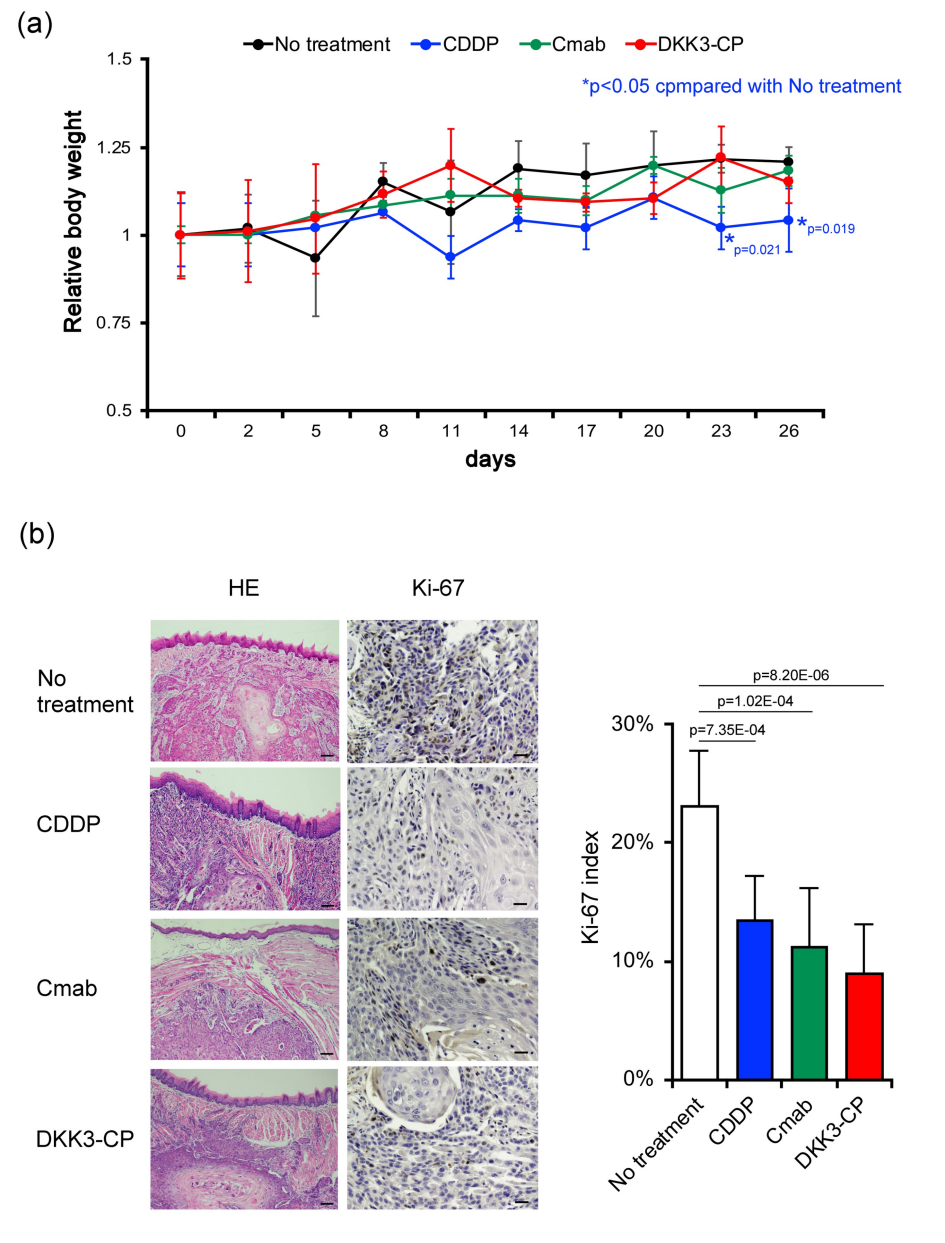
Histological evaluation of orthotopic xenograft model. (a) The weight of the mice treated with CDDP significantly decreased on day 23 and day 26 compared with the no-treatment group. The weight of the mice treated with Cmab or DKK3-CP did not show a significant change compared with the control. (b) The animal model revealed that administration of the anticancer agents significantly decreased Ki-67 indices. Scale bar 100 µm for hematoxylin-eosin (HE) and 50 µm for Ki-67. CDDP: cisplatin; Cmab: cetuximab; DKK3-CP: DKK3 complementary peptide.

The mice treated with CDDP showed significant weight loss on day 23 (p = 0.021) and day 26 (p = 0.019), compared with the "no treatment" group. On the other hand, the mice treated with Cmab or DKK3-CP did not show weight loss (Figure 4A).

In 14 mice out of 36 mice used, tumor formation was observed (control: 5; CDDP: 2; Cmab: 3; and peptide: 4). As for the tumor size, all the visible tumor masses were of similar size. Sometimes tumors were formed in the deep area of the muscle and could not be visible from the surface. Then we could not take accurate data for that. The histological findings among the groups did not show any changes and no degeneration, necrosis, or foreign body reaction, although the agents may show cytotoxic effects. The Ki-67 index revealed that all of the anticancer agents significantly suppressed tumor growth *in vivo* compared with no treatment control (CDDP: p = 7.35E-04; Cmab: 1.02E-04; and DKK3-CP: 8.220E-06), while there were no significant differences in Ki-67 indices between the groups (Figure 4B).

## Discussion

OSCC is the most common malignant tumor type of the HNSCC, and because of its high morbidity and high mortality rate, OSCC is a serious health burden in many countries [1]. Generally, cancer arises from cumulative genetic and/or epigenetic alterations, and the genetic changes in key signaling pathways have been reported so far, including PI3K/Akt/mTOR, TP53, and WNT/beta-catenin signaling, but its clinical application is still limited [10]. OSCC will be treated mainly by a surgery-based therapy followed by a combination therapy consisting of radiotherapy and chemotherapy. Surgical resection of the cancer may cause loss of tongue or gingiva with jaw bones and consequent difficulties in speech, food intake, sensuousness, and decreased quality of life [23].

As for the treatment by conventional anticancer agents, recent advancements in the optimization of the regimens have improved the anticancer effects and outcomes of OSCC treatment. But from the perspective of preventing side effects, the ideal regimen is still to be debated. CDDP is one of the key chemotherapeutic agents used in the treatment of HNSCC/OSCC, which also enhances the effectiveness of radiotherapy [24]. CDDP induces DNA crosslinking, inhibiting replication and promoting apoptosis in rapidly proliferating tumor cells, and, therefore, it may cause serious cytotoxic side effects [9]. Cmab, a monoclonal antibody against EGFR, is used for recurrence or inoperable OSCC treatment. However, it is reported that the effects of a single administration of cetuximab are reported to be limited. Although EGFR is overexpressed in over 90% of HNSCC cases, due to low EGFR mutation rates, acquired resistance, and the activation of alternative oncogenic pathways, its efficacy is insufficient [25]. Other network meta-analysis data also showed cetuximab’s overall poor performance in overall survival, progression-free survival, and locoregional control metrics, indicating its limited utility [26]. Thus, the development of a new anticancer agent is required.

We previously attempted to seek specific cancer-associated genes in HNSCC/OSCC and focused on DKK3 as a candidate [27]. DKK3 belongs to the Dickkopf WNT signaling inhibitor family, which is known as a tumor-suppressor gene because of its inhibitory function of the oncogenic WNT signaling pathway. Many reports have shown decreased expression of DKK3 in cancers, largely due to promoter hypermethylation. However, some cancer types, including HNSCC/OSCC and esophageal squamous cell carcinoma, will express DKK3. The function of DKK3 differs between DKK3-suppressed cancers and DKK3-expressing cancers; in the former, DKK3 behaves as a tumor suppressor, and in the latter, it behaves oncogenically [18,28]. Our research demonstrated that DKK3 exerts oncogenic function in HNSCC/OSCC via activation of Akt/signaling [19,20], and the two CRDs of DKK3 are necessary as functional domains for its protein-protein interaction and consequent oncogenic cellular events [21]. Then, we generated complementary peptides for DKK3 as DKK3-CP, which specifically bind to certain amino acid sequences within the CRDs and inhibit the oncogenic function of DKK3 [21]. In this research, we compared the efficacy of DKK3-CP with conventional anticancer agents, CDDP and Cmab, using two OSCC-derived cell lines.

The *in vitro* experiment results showed that DKK3-CP could significantly suppress the cancer cell viability, cellular migration, and cellular invasion. The effects of DKK3-CP were as much as those of CDDP or Cmab and showed much intense effects in cellular migration and invasion compared with CDDP. Notably, there were slight differences in the anticancer effects between HSC-3 cells and SAS cells, although these two cell lines share some characteristics, i.e., originated from human tongue cancer and have high expression of EGFR, whereas neither has a mutation and are often used as xenograft models. Overall, the *in vitro* data showed that Cmab and DKK3-CP tend to show higher anticancer effects in SAS than in HSC-3. Sasabe et al. have reported that SAS showed higher EGFR expression and higher activation of Akt than HSC-3 cells [29], which may explain why Cmab and DKK3-CP showed higher anticancer effects in SAS cells. DKK3-CP will bind to the functional domain of DKK3 protein (CRD1 and CRD2) and block the interaction between DKK3 and effector proteins and consequent activation of Akt signaling. Taken together, it is suggested that the expression and mutation status of EGFR and the activation status of Akt signaling may determine the effect of DKK3-CP.

Although the success rate was not high in this research, we could show the efficacy of DKK3-CP and compare it with that of CDDP and Cmab in the orthotopic xenograft model. The data showed that the effects of DKK3-CP were as much as conventional anticancer agents, also *in vivo*, as a pilot study. It requires discussion that the histological findings among the groups did not show any changes and no degeneration, necrosis, or foreign body reaction, although anticancer agents, particularly CDDP, may show cytotoxic effects. That may be because of the low dosage of the drugs (5 mg/kg/week) and problems in drug delivery. In this experiment, the anticancer agents were administered intraperitoneally, not by intravenous injection, since the intravenous administration of DKK3-CP was technically difficult. The ideal regimen and appropriate drug delivery should be well considered in future studies. Moreover, DKK3-CP did not show any weight loss in the animal model, suggesting the lower likelihood of unexpected side effects. However, the investigations of the detailed mechanism and assessment of possible risk by side effects in long-term administration of DKK3-CP are necessary for clinical applications.

In this study, all anticancer agents were tested as a single administration. In a future study, a combined therapeutic regimen with CDDP, Cmab, and DKK3-CP should be debated. Nevertheless, concerning unfavorable side effects of CDDP and limited effects of Cmab (which depend on overexpression status or mutation status of EGFR), the peptides may possess comprehensively slight superiority to these conventional anticancer agents.

Study limitations

This study evaluated the therapeutic effects of DKK3-CP and compared the effects with those of conventional anticancer agents (CDDP and Cmab). However, we could investigate the effect with just one regimen (5 mg/kg/week). Although we could show that DKK3-CP significantly suppressed tumor growth in this regimen as a pilot study, the ideal treatment regimen should be optimized in further investigations.

An orthotopic xenograft model was used in this study to confirm whether it can also exert anticancer effects when it is administered systemically. Although the data showed a significantly reduced Ki-67 index in histological evaluation, the results should be conservatively interpreted because of a small sample size, lack of visible tumor sizes, and no histological changes, which may be because of the low dosage of drugs.

## Conclusions

In conclusion, DKK3-CP showed significant therapeutic effects that are equivalent or partially superior to the conventional anticancer agents. DKK3-CP may be a promising new therapeutic agent for OSCC treatment. Further investigations are encouraged for the realization of clinical application of DKK3-CP.
